# Supplementary material for: RT-IVT method allows multiplex real-time quantification of in vitro transcriptional mRNA production
Source: Commun Biol. 2023 Apr 24;6:453. doi: 10.1038/s42003-023-04830-1 (PMC10124930; doi:10.1038/s42003-023-04830-1)
Supplement: Supplementary file 2 — Supplementary information [file 42003_2023_4830_MOESM2_ESM.pdf]

Supporting Information for

RT-IVT method allows multiplex real-time quantification of *in vitro* transcriptional mRNA production

Fengyu Zhang[1#], Yipeng Wang[1#], Xiaomeng Wang[1], Hongjie Dong[2], Min Chen[1], Ning Du[3], Wei Hu[1], Kundi Zhang[1*], Lichuan Gu[1*]

[1]State Key Laboratory of Microbial Technology, Shandong University, 72 Binhai Road, Qingdao 266237, China

[2]Shandong Institute of Parasitic Diseases, Shandong First Medical University & Shandong Academy of Medical Sciences, 11 Taibaizhong Road, Jining 272033, China

[3]Institute of Ecology and Biodiversity, School of Life Sciences, Shandong University, 72 Binhai Road, Qingdao 266237, China

[#]These authors contributed equally.

*Corresponding Author:

Lichuan Gu
Tel.: 0532-58632443
Email:lcgu@sdu.edu.cn

Kundi Zhang
Tel.: 0532-58632445
Email: kdzhang@sdu.edu.cn

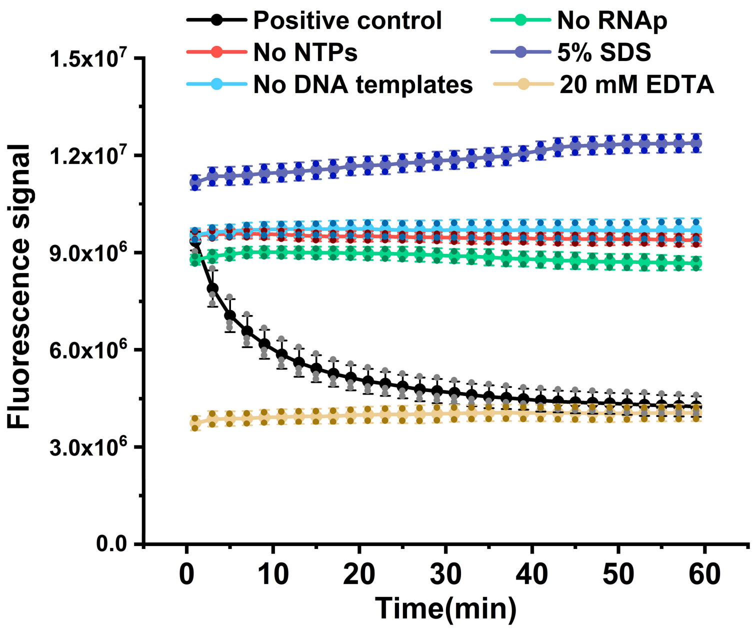

Supplementary Figure S1. Comparison of negative control groups in RT-IVT assays. 200 nM T7 RNA polymerase, 30 nM DNA template 1, 250 μM NTP mixture, 500 nM BFQ1 and 50 nM PBCV-1 DNA ligase were mixed in transcription buffer and tested in a RT-PCR thermocycler. The values shown were the mean ± standard deviation of *n* = 2–3 replicates.

Supplementary Table S1. Complementary RNA, DNA and templates used in this study.

cDNA & Template DNA	Sequence (5′ to 3′)
cRNA	CGCCGUCAACCACCAUCCAAAAAGAAAAACGCCUG CUGGGGCAA
cDNA 1	CGCCGTCAACCACCATCCAAAAAGAAAAACGCCTGC TGGGGCAA
cDNA 2	GCCGAACTCAGAAGTGAAACGCCGTAGAGACGATG GTAGTGTGGGGTCTCCC
cDNA 3	ATACCTGCTGCCGACCGCTGCTGCAAGCATGCTGCT CCTCGCTGCCCA
DNA template 1	CCATACCCACGCCGAAACAAGCGCTCATGAGCCCG AAGTGGCGAGCCCGATCTTCCCCATCGGTGATGTC GGCGATATAGGCGCCAGCAACCGCACCTGTGGCGC CGGTGATGCCGGCCACGATGCGTCCGGCGTAGAGG ATCGAGATCTCGATCCCGCGAAATTAATACGACTCAC TATAGGGGAATTGTGAGCGGATAACAATTCCCCTCTA GAAATAATTTTGTTTAACTTTAAGAAGGAGATATACAT ATGAACGCCGTCAACCACCATCCAAAAAGAAAAACG CCTGCTGGGGCAAGCCGGCGATGGCCATGGATATC GG
DNA template 2	CCATACCCACGCCGAAACAAGCGCTCATGAGCCCG AAGTGGCGAGCCCGATCTTCCCCATCGGTGATGTC GGCGATATAGGCGCCAGCAACCGCACCTGTGGCGC

	CGGTGATGCCGGCCACGATGCGTCCGGCGTAGAGG<br>ATCGAGATCTCGATCCCGCGAAATTAATACGACTCAC<br>TATAGGGGAATTGTGAGCGGATAACAATTCCCCTCTA<br>GAAATAATTTTGTTTAACTTTAAGAAGGAGATATACAT<br>ATGAAGCCGAACTCAGAAGTGAAACGCCGTAGAGA<br>CGATGGTAGTGTGGGGTCTCCCGCCGGCGATGGCC<br>ATGGATATCGG
DNA template 3	CCATACCCACGCCGAAACAAGCGCTCATGAGCCCG<br>AAGTGGCGAGCCCGATCTTCCCCATCGGTGATGTC<br>GGCGATATAGGCGCCAGCAACCGCACCTGTGGCGC<br>CGGTGATGCCGGCCACGATGCGTCCGGCGTAGAGG<br>ATCGAGATCTCGATCCCGCGAAATTAATACGACTCAC<br>TATAGGGGAATTGTGAGCGGATAACAATTCCCCTCTA<br>GAAATAATTTTGTTTAACTTTAAGAAGGAGATATACAT<br>ATGAAATACCTGCTGCCGACCGCTGCTGCAAGCATG<br>CTGCTCCTCGCTGCCCAGCCGGCGATGGCCATGGA<br>TATCGG
DNA template$^{\text{BFQ1\&Binary probes}}$	CCATACCCACGCCGAAACAAGCGCTCATGAGCCCG<br>AAGTGGCGAGCCCGATCTTCCCCATCGGTGATGTC<br>GGCGATATAGGCGCCAGCAACCGCACCTGTGGCGC<br>CGGTGATGCCGGCCACGATGCGTCCGGCGTAGAGG<br>ATCGAGATCTCGATCCCGCGAAATTAATACGACTCAC<br>TATAGGGGAATTGTGAGCGGATAACAATTCCCCTCTA<br>GAAATAATTTTGTTTAACTTTAAGAAGGAGATATACAT<br>ATGAACGCCGTCAACCACCATCCAAAAAGAAAAACG<br>CCTGCTGGGGCAAACTGAGCCCATTGGTATCGTGG<br>AAGGACTCGCCGGCGATGGCCATGGATATCGG
DNA template $^{\textit{fhuFp}}$	AATCCGCCACTGATCTGACGATAAATCCGCATACATC<br>AGCCCTGCAATCAGCAATCCCGGCAGCAACACTCC<br>CCAGCCACTGCCCAGCGTACGTTGCAACATGATTTC<br>ATCTCTTTCATTGATAATGATAACCAATATCATATGATA<br>ATTTTTATCATTTGCAAGCCAGATAAATCCCTTGCTAT<br>CGGGTAAACCTATCGCTATGATTAGCAATCATTATCAT<br>TTAGATTACTATCCCGATTCGCCGTCAACCACCATCC<br>AAAAAGAAAAACGCCTGCTGGGGCAAACCAGCGTG<br>GACCGCTTGCTGCAACTCTCTCA
DNA template^{Fur}	CCCGCGAAATTAATACGACTCACTATAGGGATAATGA<br>TAATGATAATGATAATGATAATGATGTGAGCGGATAA<br>CAATTCCCCTCTAGAAATAATTTTGTTTAACTTTAAG<br>AAGGAGATATACATATGAACGCCGTCAACCACCATC<br>CAAAAAGAAAAACGCCTGCTGGGGCAAGCCGGCGA<br>TGGCCATGGATATCGGA

Supplementary Table S2. Primers used in this study.

Primer	Sequence (5′ to 3′)
DNA template 1-F	CCATACCCACGCCGAAACAA
DNA template 1-R	CCGATATCCATGGCCATCGCCGGCTTGCCCCAGCAGGCGTTTTTCTTTTTGGATGGTGGTTGACGGCGTTCATATGTATATCTCCTTCTTAAAGTTAAAC
DNA template 2-R	CCGATATCCATGGCCATCGCCGGCGGGAGACCCCACACTACCATCGTCTCTACGGCGTTTCACTTCTGAGTTCGGCTTCATATGTATATCTCCTTCTTAAAGTTAAAC
DNA template 3-R	CCGATATCCATGGCCATCGCCGGCTGGGCAGCGAGGAGCAGCATGCTTGCAGCAGCGGTCGGCAGCAGGTATTTCATATGTATATCTCCTTCTTAAAGTTAAAC
DNA template$^{BFQ1\&Binary\ probes}$-R	CCGATATCCATGGCCATCGCCGGCGAGTCCTTCCACGATACCAATGGGCTCAGTTTGCCCCAGCAGGCGTTTTTCTTTTTGGATGGTGGTTGACGGCGTTCATATGTATATCTCCTTCTTAAAGTTAAAC
DNA template ^{fhuFp}-F	AATCCGCCACTGATCTGACG
DNA template ^{fhuFp}-R	TGAGAGAGTTGCAGCAAGCGGTCCACGCTGGTTTGCCCCAGCAGGCGTTTTTCTTTTTGGATGGTGGTTGACGGCGAATCGGGATAGTAATCTAAATGATAATG
qRT-PCR-F	AGCGGATAACAATTCCCCTCT
qRT-PCR-R	GCTTGCCCCAGCAGGCGT
