# Supplementary material for: RT-IVT method allows multiplex real-time quantification of in vitro transcriptional mRNA production
Source: Commun Biol. 2023 Apr 24;6:453. doi: 10.1038/s42003-023-04830-1 (PMC10124930; doi:10.1038/s42003-023-04830-1)
Supplement: Supplementary file 3 — Description of Additional Supplementary Files [file 42003_2023_4830_MOESM3_ESM.pdf]

Description of Additional Supplementary Files

File name: Supplementary Data 1

Description: The source data behind the graphs in the paper
